# Excess weight and abdominal obesity in postmenopausal Brazilian women: a population-based study

**DOI:** 10.1186/1472-6874-13-46

**Published:** 2013-11-14

**Authors:** Angela A F Gravena, Sheila C R Brischiliari, Tiara C R Lopes, Cátia M D Agnolo, Maria D B Carvalho, Sandra M Pelloso

**Affiliations:** 1 Department of Health Science, Universidade Estadual de Maringa, Maringa, Parana, Brazil; 2 Department of Nursing, Universidade Estadual de Maringá, Maringa, Parana, Brazil; 3 Department of Medicine, Universidade Estadual de Maringá, Maringa, Parana, Brazil

**Keywords:** Postmenopause, Body mass index, Waist circumference, Obesity, Abdominal obesity

## Abstract

**Background:**

The menopause is associated with a tendency to gain weight. Several alterations in fat deposits occur, leading to changes in the distribution of body fat. There are strong indications that, in middle age, obesity is associated with increased mortality. This study set out to determine the factors associated with the prevalence of overweight and abdominal obesity in postmenopausal women in a population-based study in Brazil.

**Methods:**

The sample included 456 women, aged 45–69 years, residing in the urban area of Maringa, Parana. Systematic sampling, with a probability proportional to the size of the census sector, was performed. Behavioral, economic, and sociodemographic data were collected, and body mass index (BMI) and waist circumference (WC) were determined.

**Results:**

According to BMI criteria (≥25.0 kg/m^2^), 72.6% of the women were overweight, and according to WC (≥88 cm), 63.6% had abdominal obesity. Based on logistic regression analysis, the factors that were most closely associated with overweight were: having three or more children (odds ratio (OR): 1.78; 95% confidence interval (CI): 1.06–3.00); and not taking hormone replacement therapy (OR: 1.69; 95% CI: 1.06–2.63). The prevalence of abdominal obesity was positively associated with greater parity (OR: 1.34, 95% CI: 1.05–1.72) and age older than 65 years (OR: 1.50; 95% CI: 1.03–2.19).

**Conclusions:**

This study found that the prevalences of overweight and abdominal obesity were higher for postmenopausal women who had three or more children. Age over 65 years was also a risk factor for abdominal obesity and no use of hormonal replacement therapy was a risk factor for overweight.

## Background

The incidence of excess body weight among women is increasing worldwide to epidemic proportions [1]. The arrival of the menopause in middle age is associated with a tendency to gain weight [2,3]. It is estimated that the prevalence of obesity among women aged 40–59 years in the United States is approximately 38.2%, while the prevalence of overweight and obesity is 66.3% [4]. In Brazil, data from the Family Budget Study (Pesquisa de Orçamento Familiar, POF) revealed that 58.0% and 63.0% of women aged 45–54 and 55–64 years of age, respectively, were overweight, and, of these, 21.5% and 26.0%, respectively, were obese [5]. There are strong indications that in middle age, obesity is associated with increased mortality [6].

Several alterations in fat deposits occur with the advent of the menopause, leading to a change in the distribution of body fat. Hypoestrogenism has a negative effect on fat metabolism, favoring the appearance of central-body obesity [7,8]. The North American Menopause Society [2] states that aging, lifestyle, and behavioral factors, such as a lack of exercise and increase in food consumption, are closely linked with weight gain in the menopause. Several studies have been conducted in an attempt to characterize the factors associated with obesity in the menopause [9-12]; however, as far as we are aware, there are few published population-based studies which have evaluated the factors associated with overweight and abdominal obesity in postmenopausal women.

A retrospective study based on an analysis of hospital charts associated postmenopausal obesity with older age, greater parity, a less intense smoking habit, a shorter menopausal period, and a surgical menopause [11]. Among women who were seen in outpatient clinics, the proportion with abdominal obesity was higher among those with 7 years or less of schooling, and who were sedentary or insufficiently active [10]. One population-based study performed in Brazil, which was carried out in the municipality of Rio de Janeiro in 1996, concluded that the association between the menopause and overweight was not linked with age or physical inactivity [13]. Only population-based health studies make it possible to collect data to determine the indicators associated with health and disease, as well as the risk factors and social determinants of the health/disease process [14].

In view of these considerations, the objective of this study was to evaluate the factors associated with the prevalence of overweight and abdominal obesity in postmenopausal women in Brazil, by means of a population-based study.

## Methods

This cross-sectional, population-based study included women aged 45–69 years, residing in the urban area of the municipality of Maringa, state of Paraná, Brazil, between December 2010 and June 2011. The women had ceased menstruation for at least 12 months and had a natural menopause. This latter criterion was established since women who had a hysterectomy would not have undergone the temporal hormonal and psychological changes typical of the climacteric period. Women with insulin-dependent diabetes, uncontrolled hypertension or diseases of the thyroid were excluded from the study. The study was approved by the Standing Committee on Ethics in Research on Human Beings of the Federal University of Maringa (Protocol No. 201/2010).

The sample size was estimated with the objective of ensuring that it was representative of the population. It was calculated using data of the female population of the Municipality of Maringa aged 45–69 years in the Demographic Census reported by the Brazilian Institute of Geography and Statistics (Instituto Brasileiro de Geografia e Estatistica, IBGE) [15] in the year 2000, and the projected population in the year 2009, which totaled 36,023 women. Using a confidence interval (CI) of 95% (z =1.96), the estimate of the sample should be within ± 5% (e) of the real proportion of the population of women in this age range. The statistical model n = z^2^[p(1-p)]:e^2^ for infinite populations (N > 10,000) was used, which provided the sample size, in this case 380 women. To calculate the sample, the statistics program Epi-Info version 3.5.1. (CDC, Atlanta, GA, USA) was used, with a CI of 0.95 and a margin of error of 0.05. With the addition of 20%, in case of possible losses and/or refusals to participate, the total sample size was 456 women.

For the present study, the reference unit for sample selection was the census sectors of the municipality, according to IBGE; the primary sampling unit was the census sectors, and the secondary units were the households. A systematic sampling strategy was used, with the probability proportional to the size of the sector.

The Municipality of Maringa contains, according to IBGE, 407 census sectors, and 368 sectors characterized as urban areas were included in this study. In order to obtain a representative randomness, systematic sampling was used to select the households. From a map of the blocks enumerated in each census sector, the starting point of the survey as well as the start of each sector were randomly selected, with movement in a clockwise direction. For each sector, a simple random sample proportional to the number of women residing in each of these sectors was selected to obtain the total sample size (N = 456).

Because the number of women to be interviewed in each sector was proportional to its size, and to best compensate for any effect of the neighborhood, one household was randomly selected, and the next three were skipped. If no woman resided in the house selected, the interviewer moved to the next, and this process was repeated for each interview. If more than one eligible woman lived in the home, one was randomly chosen for interview.

The home visits included the use of a face-to-face questionnaire and measurement of anthropometric parameters (weight, height, and waist size) after informed consent had been obtained from the subject. In order to standardize the procedure of taking objective measurements, all the field researchers received identical training. The questionnaire was pre-tested on 30 women in the same age range as in the study, in the area of a Local Health Unit (Unidade Local de Saúde) of the municipality. A pilot study was carried out with 30 subjects in one census sector, which was randomly selected from among the sectors that were not included in the study itself.

The primary variables evaluated in the study were body mass index (BMI) and waist circumference (WC). Body weight and height were recorded in duplicate, and the BMI was calculated later to determine the women’s current weight class based on the World Health Organization [16] definition of overweight as BMI ≥25.0 kg/m^2^, and obesity as BMI ≥30.0 kg/m^2^. For the purposes of the present evaluation, WC ≥88 cm was considered to indicate the presence of abdominal obesity [17].

The secondary measurements or independent variables evaluated were: age (calculated in full years on the date of the interview and stratified into three groups: 45–54; 55–64 and 65–69 years); age at menopause (defined as the age at last menstruation as reported by the interviewee); skin color (white or nonwhite); degree of schooling (up to 7 years, or 8 or more years); marital status (with or without a partner); family income and class (economic class A: average income R$ 8148.00; B: average income R$ 2746.00; C: average income R$ 960.00; D: average income R$ 485.00; or E: average income R$ 277.00, according to the economic classification criteria used in Brazil) [18]; occupation (presence or absence of paid work); parity (0, 1, 2, or 3 or more births); level of physical activity (according to the criteria of the Brazilian Society of Cardiology [19], sedentary: no regular physical activity, or regular physical activity, i.e., a minimum frequency of three times a week for at least 30 minutes of any type of exercise); smoking (daily smoking habit independent of quantity, or no smoking); use of hormone replacement therapy (no hormone therapy in the last 6 months, or continuous hormone therapy for at least 6 months).

In the statistical analysis, the odds ratio (OR) for the association of independent variables with overweight and abdominal obesity was calculated using the program Epi Info 3.5.1. In the next step, the variables with p < 0.20 were selected for multivariate analysis by logistic regression to evaluate the independent variables associated with overweight and abdominal obesity, using the program Statistica 7.1 (StatSoft, Tulsa, OK, USA) and a 95% significance level.

## Results

The mean (± standard deviation) age of the 456 women was 58.7 ± 5.7 years, and the mean age at onset of the menopause was 48.0 ± 5.0 years. The socioeconomic, demographic and behavioral are presented in Table 1.

**Table 1:** Distribution of the behavioral, demographic, and socioeconomic variables, prevalence of excess weight, odds ratios (OR), and confidence intervals (95% CI) in postmenopausal women in Maringa, Parana, Brazil, 2011

**Variables**	**no. (%)**	**Prevalence of excess weight**^**a**^	**OR (95% CI)**	**p**
**Age (years)**				
45-54	110 (24.1)	68.2	1.0	
55-64	260 (57.0)	73.8	1.32 (0.79–2.21)	0.17
65-69	86 (18.9)	74.4	1.36 (0.69–2.67)	0.24
**Years of schooling**				
< 8	258 (56.6)	77.5	1.76 (1.14–2.37)	< 0.01
≥8	198 (43.4)	66.2	1.0	
**Marital status**				
With partner	288 (63.2)	75.3	1.45 (0.93–2.25)	0.08
Single	168 (36.8)	67.9	1.0	
**Skin color**				
White	388 (85.1)	73.2	1.0	
Nonwhite	68 (14.9)	69.1	1.22 (0.89–1.26)	0.48
**Social class**				
A and B	179 (39.3)	69.8	1.0	
C and D	277 (60.7)	74.4	1.25 (0.81–1.94)	0.28
**Occupation**				
Paid	242 (53.1)	67.8	1.0	
Unpaid	214 (46.9)	78.0	1.69 (1.09–2.63)	0.01
**Parity**				
0, 1 or 2	186 (40.8)	65.1	1.0	
3 or more	270 (59.2)	77.8	1.88 (1.21–2.91)	< 0.01
**Physical activity**				
Sedentary	327 (71.7)	72.5	0.98 (0.60–1.59)	0.93
Active	129 (28.3)	72.9	1.0	
**Smoker**				
Yes	55 (12.1)	65.5	1.0	
No	401 (87.9)	73.6	0.93 (0.36–1.39)	0.21
**Hormone therapy use**				
Yes	84 (18.4)	63.1	1.0	
No	372 (81.6)	74.7	1.73 (1.02–2.94)	0.03

^a^Body mass index ≥ 25.0 kg/m^2^.

The mean BMI of the women was 28.6 ± 5.5 kg/m^2^, 72.6% were overweight, and 35.5% were obese. The mean WC was 99.8 ± 9.9 cm, and 63.6% had abdominal obesity.

The univariate analysis initially demonstrated a significant association between overweight and years of schooling, occupation, parity, and hormonal therapy. The prevalence of overweight increased in women with less schooling (p < 0.01), with no paid occupation (p = 0.01), and who had three or more children (p < 0.01). In contrast, the use of hormone therapy was associated with a lower prevalence of overweight (p = 0.04) (Table 1).

The prevalence of abdominal obesity was positively associated with age and parity. For women older than 65 years or who had three or more children, the prevalence of abdominal obesity was 74.4% and 68.5%, respectively (p < 0.01) (Table 2).

**Table 2:** Distribution of the prevalence of abdominal obesity according to the behavioral, demographic, and socioeconomic variables, OR and 95% CI

**Variables**	**Prevalence of abdominal obesity**^**a**^	**OR**	**p**
		**(95% CI)**	
**Age (years)**			
45–54	56.4	1.0	
55–64	63.1	1.32 (0.82–2.13)	0.22
65–69	74.4	2.25 (1.17–4.36)	< 0.01
**Years of schooling**			
< 8	65.9	1.26 (0.84–1.88)	0.24
≥8	60.6	1.0	
**Marital status**			
With partner	65.3	1.22 (0.81–1.84)	0.32
Single	60.7	1.0	
**Skin color**			
White	63.1	1.0	
Nonwhite	66.2	1.14 (0.64–2.04)	0.63
**Social class**			
A and B	61.5	1.0	
C and D	64.9	1.16 (0.77–1.75)	0.44
**Occupation**			
Paid	59.9	1.0	
Unpaid	67.8	1.41 (0.94–2.11)	0.08
**Parity**			
0, 1 or 2	56.5	1.0	
3 or more	68.5	1.68 (1.12–2.52)	< 0.01
**Physical activity**			
Sedentary	65.4	1.32 (0.85–2.05)	0.19
Active	58.9	1.0	
**Smoker**			
Yes	56.4	1.0	
No	64.6	1.41 (0.77–2.59)	0.23
**Hormone therapy use**			
Yes	57.1	1.0	
No	65.1	1.40 (0.84–2.82)	0.17

^a^Waist circumference ≥ 88 cm.

The logistic regression analysis demonstrated an association of overweight with parity and lack of hormonal therapy. Women with three or more children and non-users of hormonal therapy showed, respectively, 1.78 (95% CI: 1.06–3.00) and 1.69 (95% CI: 1.09–2.63) times higher risk of being overweight (Table 3). The multivariate analysis also found that the prevalence of abdominal obesity had a positive association with women having three or more children (OR: 1.34; 95% CI: 1.05–1.72) and with age older than 65 years (OR: 1.50; 95% CI: 1.032.19) (Table 4).

**Table 3:** Multivariate analysis for the prevalence of overweight in postmenopausal women according to the variables included in the model

**Variable**	**Adjusted OR**	**95% CI**	**p**
**Age (years)**			
55–64	1.20	0.71–2.01	0.28
65–69	1.00	0.51–1.97	0.37
**Marital status** (with partner)	1.43	0.92–2.23	0.10
**Years of schooling** (< 8)	1.43	0.91–2.25	0.11
**Occupation** (unpaid)	1.39	0.88–2.17	0.14
**Parity** (3 or more)	1.78	1.06–3.00	0.02
**Hormone therapy use** (no)	1.69	1.09–2.63	0.01

**Table 4:** Multivariate analysis for the prevalence of abdominal obesity in postmenopausal women, according to the variables included in the model

**Variable**	**Adjusted OR**	**95% CI**	**p**
**Age (years)**			
55-64	1.18	0.89–1.57	0.24
65-69	1.50	1.03–2.19	0.03
**Occupation** (unpaid)	1.17	0.92–1.50	0.18
**Parity** (3 or more)	1.34	1.05–1.72	0.01
**Hormone therapy use** (no)	1.22	0.90–1.65	0.19
**Physical activity** (sedentary)	1.18	0.91–1.54	0.19

## Discussion

In this study, after adjustment of the variables, greater parity (three or more children) was associated with overweight and abdominal obesity. Age over 65 years was a risk factor for abdominal obesity and no use of hormonal replacement therapy was a risk factor for overweight.

The gestational experience is a stage of a woman’s lifecycle that contributes to a temporary change in body composition, with a gain in weight. In the midst of the nutritional transition, physiological factors inherent to the gestational period increase the individual’s predisposition to excessive weight gain during gestation, making the woman susceptible to the development of obesity [20]. The accumulation of fat, resistance to insulin, and secretion of glucocorticoids observed during pregnancy, as well as the reduction in ovulation cycles in multiparous women, may be factors in the correlation between obesity and parity. In addition, one must consider that maternal-care activities produce changes in physical activity, diet, and social habits [21].

The finding in this study of an association between overweight and parity concords with the results of an investigation carried out in 508 Chilean women, which found a positive relationship between parity and BMI, leading to a body-weight increase of 0.46 kg for each child born [22]. A retrospective analysis of the medical records of 574 postmenopausal Spanish women observed a positive correlation between obesity and the number of children born [11]. Among studies in Brazil, an investigation carried out in São Paulo in postmenopausal women showed that 42.2% of those with three or more children were obese [10].

With respect to the analysis of abdominal obesity and parity, the literature shows that multiparous women have larger values of WC [23]. Lassek and Gaulin [21] determined that WC was 3.26 cm greater in multiparous compared with nulliparous women, and also found that parity was associated with a relative decrease in hip circumference and an increase in WC after adjustment for age and BMI.

In relation to age and abdominal obesity, a longitudinal 9-year study with 949 participants investigated the natural history of the menopausal transition and showed that WC increased with aging and accelerated after the menopause [24]. The increase in WC with age was largely driven by gains in body weight, but increases in WC have also been reported with aging in the absence of weight gain [25].

The finding that non-use of hormonal therapy was associated with overweight and obesity merits attention, because it is a common perception among women that the use of hormonal therapy can cause overweight. The present study found a higher proportion of women with overweight in the group who did not use hormone replacement therapy. These findings are similar to those of other investigations, which concluded that there was no significant evidence that hormonal therapy caused a weight increase in postmenopausal women [9,10]. A 1-year controlled study to evaluate the effect of tibolone on body composition in postmenopausal women concluded that there was a decrease in fat mass and an increase in muscle mass in individuals treated with the hormone compared with a control group. The changes related to an increase in muscle mass were attributed to the androgenic properties of tibolone [26].

A reduction in ovarian hormones at the menopause leads to diverse functional and endocrinological disturbances, among them an increase in body weight and a decrease in basal metabolism, which leads to greater weight gain [2,27]. Another factor related to the control of body weight is the effect of activation of estrogen receptors. Estrogen receptor-α, activated by estradiol, has a crucial role in inhibiting the development of adipose tissue; therefore, there is an increase in adipose tissue during the menopause as a result of the deficiency of estrogen, and this can be alleviated by hormone replacement therapy [28]. A longitudinal study that investigated the effect of the change in reproductive hormones during the menopause on incident obesity (BMI ≥30 kg/m^2^) and severe obesity (BMI ≥35 kg/m^2^) demonstrated that a decrease in sex hormone-binding globulin over time was strongly associated with both incident obesity and severe obesity [29].

A review that aimed to summarize the literature regarding the impact of the menopause on body weight and body composition concluded that the tendency towards greater central abdominal fat accumulation is ameliorated by estrogen therapy [30]. The North American Menopause Society states that hormonal therapy, regardless of the type (estrogen or estrogen-progestogen), does not cause overweight [2]. If overweight is associated with the menopause, it is important to consider that several different factors may be involved in increasing body weight during this phase of a woman’s life, among them the direct effect of hormonal reduction, changes in mood, depression, sedentary habits, inappropriate eating habits, and heredity [2,31].

One limitation was the cross-sectional design of the study. A second limitation was the factor of hormonal therapy use, with no distinction between types and routes of administration. We also did not consider changes in mood, depression, and factors related to lifestyle that can have an impact on obesity [32,33]. Another limitation was that there was no evaluation of nutritional state prior to the menopause, which is among the determinants of the nutritional state in older age [34].

## Conclusions

This study indicated that, in an urban population of Brazilian women, overweight and abdominal obesity were associated with greater parity, a lack of hormonal therapy and age over 65 years. To our knowledge, this is one of the few population-based studies with the objective of investigating the factors related to obesity in postmenopausal women. These results may have important implications for women’s health, since overweight and abdominal obesity can lead to the development of chronic diseases, including cardiovascular diseases, and significantly increase the costs of hospitalization. However, factors that operated prior to the onset of menopause should be studied and evaluated, including nutritional state, eating habits, smoking, and depression. Further analyses are needed to evaluate these points, ideally through longitudinal prospective studies.

## Competing interests

The authors declare that they have no competing interests.

## Authors’ contributions

AAFG, SCRB, TCRL, CMDA participated in the design of the study, performed the statistical analysis, conceived the study, participated in its design and coordination, and helped draft the manuscript. MDBC and SMP participated in study design and coordination and helped draft the manuscript. All authors read and approved the final manuscript.

## Pre-publication history

The pre-publication history for this paper can be accessed here:

http://www.biomedcentral.com/1472-6874/13/46/prepub
